# Microarray profiling of circular RNAs in human papillary thyroid carcinoma

**DOI:** 10.1371/journal.pone.0170287

**Published:** 2017-03-13

**Authors:** Nianchun Peng, Lixin Shi, Qiao Zhang, Ying Hu, Nanpeng Wang, Hui Ye

**Affiliations:** 1 Department of Endocrinology and Metabolism, Affiliated Hospital of Guizhou Medical University, Guiyang, China; 2 Department of Thyroid Surgery, Affiliated Hospital of Guizhou Medical University, Guiyang, China; University of Toronto, CANADA

## Abstract

**Background:**

Non-coding circular RNAs (circRNAs) have displayed dysregulated expression in several human cancers. Here, we profiled the circRNA expression of papillary thyroid carcinoma (PTC) tumors to improve our understanding of PTC pathogenesis.

**Methods:**

Microarray profiling was performed on 18 thyroid samples, consisting of six PTC tumors, six matching contralateral normal samples, and six benign thyroid lesions. After low-intensity filtering, hierarchical clustering revealed the circRNA expression patterns. Statistical analysis followed by qRT-PCR validation identified the differential circRNAs. MicroRNA (miRNA) target prediction software identified putative miRNA response elements (MREs), which were used to construct a network map of circRNA-miRNA interactions for the differential circRNAs. Bioinformatics platforms predicted cancer-related circRNA-miRNA associations and putative downstream target genes, respectively.

**Results:**

A total of 88 circRNAs and 10 circRNAs were significantly upregulated and downregulated, respectively, in PTC tumors relative to normal thyroid tissue, while 129 circRNAs and 226 circRNAs were significantly upregulated and downregulated, respectively, in PTC tumors relative to benign thyroid lesions. A total of 12 upregulated and four downregulated circRNAs were overlapping between the foregoing comparisons. One downregulated circRNA (hsa_circRNA_100395) showed interactive potential with two cancer-related miRNAs (miR-141-3p and miR-200a-3p). From this analysis, we identified several promising cancer-related genes that may be targets of the dysregulated hsa_circRNA_100395/miR-141-3p/miR-200a-3p axis in PTC tumors.

**Conclusions:**

circRNA dysregulation may play a role in PTC pathogenesis, and several key circRNAs show promise as candidate biomarkers for PTC. The hsa_circRNA_100395/miR-141-3p/ miR-200a-3p axis may be involved in the pathogenesis of PTC.

## Introduction

Thyroid carcinoma is the most common endocrine cancer with most countries showing mortality rates of 0.2–0.4 per 100,000 men and 0.2–0.6 per 100,000 women [1]. Most countries have displayed a rising incidence of thyroid cancer (mainly papillary carcinomas) over the past several decades, which has been attributed to improved diagnostic techniques over this time period [1]. Despite these improved diagnostic methods, the gold standard technique—fine-needle aspiration (FNA) cytology–only yields determinate findings at a ~70% success rate [2]. Therefore, in order to reduce the rate of invasive and costly diagnostic thyroidectomies, the development of alternative non-invasive diagnostic approaches as adjuncts to FNA cytology remains a pressing clinical challenge [2].

To this end, the dysregulated expression of non-coding, single-stranded RNAs termed microRNAs (miRNAs, miRs) have been closely associated with the pathogenesis of human cancers, as miRNAs have been shown to regulate cellular phenomena associated with oncogenesis, including cellular differentiation, adhesion, and apoptosis [2]. With respect to thyroid cancer, several miRNAs (i.e., miR-220, miR-221, and miR-222) have been shown to be significantly upregulated, while several other miRNAs (i.e., let-7, miR-26, and miR-345) have been shown to be significantly downregulated in papillary thyroid carcinoma (PTC) cells [3–6].

Although miRNAs have demonstrated promise as molecular biomarkers for cancer, miRNAs are not the only type of non-coding RNAs that have been shown to regulate gene expression in cancer cells [7]. Circular RNAs (circRNAs) are a newly-discovered type of non-coding RNA that are formed from the covalent linkage of the 3’ and 5’ ends to form a closed loop [8]. As a result of this closed structure, circRNAs have been shown to be highly stable and largely resistant to RNA degradative pathways [9], which suggests that circRNAs may be more technically suitable as molecular biomarkers for human cancers. Similar to miRNAs, several circRNAs have shown dysregulated expression in human cancers. For example, the expression of cir-ITCH (hsa_circ_0001141, hsa_circ_001763) has been shown to be significantly downregulated in squamous cell carcinoma of the esophagus as well as colorectal cancer tumors, the expression of hsa_circ_002059 has been shown to be significantly downregulated in gastric malignancies, and the expression of hsa_circ_0001649 has been shown to be significantly downregulated in hepatocellular carcinoma (HCC) tumors [9].

Despite these promising circRNA findings across various types of human cancers, no study has yet profiled circRNA expression in human PTC. Therefore, here we profiled the circRNA expression of PTC tumors in order to improve our understanding of the pathogenesis of PTC as well as to identify potential circRNA biomarkers for PTC.

## Methods

### Ethics statement

This study was approved by the Ethics Committee of the Affiliated Hospital of Guizhou Medical University (approval no.: 2014(92), Guiyang, China). All subjects recruited for this study provided written informed consent prior to participation.

### Tumor specimen collection

From October 2015 to December 2015, thyroid tissue samples were collected from consecutively recruited patients that underwent thyroidectomy at the Affiliated Hospital of Guizhou Medical University (Guiyang, China). The specimens were immediately snap-frozen in liquid nitrogen post-resection and then refrigerated at -80°C. Histopathological analysis of all thyroid tissue specimens was independently performed by two licensed pathologists to validate the quality of the specimens. After histopathological vetting, a total of 18 thyroid samples–consisting of six PTC tumors, six matching contralateral normal samples, and six benign thyroid lesions (i.e., three follicular adenoma samples and three multinodular goiter samples)–were finally included in the study.

### RNA isolation

All of the following RNA isolation protocols were performed in an RNA-dedicated work area with RNase/DNase-free water and RNase-free labware. Total RNA was extracted from the thyroid samples with TRIzol reagent (Invitrogen, Carlsbad, CA, USA) according to the kit’s instructions. The resulting RNA pellet was washed in 75% ethanol (1 ml) twice, air-dried, and re-suspended in RNAse/DNase-free water (20 μl). Turbo DNase Kit (Ambion) was then applied to the RNA solution to degrade any remaining DNA. The RNA was then purified and concentrated with an RNeasy MinElute Cleanup kit (Qiagen, Valencia, CA, USA). The total RNA concentrations and quality of each sample were then assessed with a NanoDrop ND-1000 spectrophotometer (NanoDrop, Wilmington, DE, USA). Specifically, OD260/OD280 ratios between 1.8 and 2.1 deemed acceptable, while OD260/OD230 ratios of greater than 1.8 were deemed acceptable. RNA integrity and DNA contamination were then assessed through electrophoresis on a denaturing agarose gel.

### Sample labeling and microarray hybridization

The Arraystar Human circRNA Microarray was used to perform the sample labeling and microarray hybridization according to the kit's instructions (Arraystar, Rockville, Maryland, USA). Briefly, each RNA sample was first treated with Ribonuclease R (RNase R; Epicenter, Madison, WI, USA) in order to degrade any linear RNA and enrich circRNA. Then, the Super RNA Labeling Kit (Arraystar) was used to amplify and transcribe the circRNAs into fluorescently-labeled cRNAs through random priming. The fluorescently-labeled cRNAs were then purified with an RNeasy Mini Kit (Qiagen). The concentrations of fluorescently-labeled cRNAs of each sample (pmol Cy3 per μg cRNA) were then quantified with a NanoDrop ND-1000 spectrophotometer (NanoDrop). Based on these calculated concentrations, 1 μg of each sample’s fluorescently-labeled cRNA was fragmented by treatment with 10× Blocking Agent (5 μl) and 25× Fragmentation Buffer (1 μl) for 30 minutes at 60°C. Then, 2× Hybridization Buffer (25 μl) was added to the fluorescently-labeled cRNA. The resulting hybridization solution (50 μl) was placed in a gasket slide, which was then assembled with the Arraystar Human circRNA Microarray slides according to the kit’s instructions (Arraystar). The slides were then incubated in an Agilent Hybridization Oven for 17 hours at 65°C. The hybridized microarrays were washed, fixed, and scanned with an Agilent G2505C Scanner (Agilent).

### Microarray data analysis

The microarray data has been deposited in the Gene Expression Omnibus (GEO) database (accession number GSE93522 [NCBI tracking system #18224128]). Agilent Feature Extraction software (version 11.0.1.1, Agilent) was used to acquire and analyze the scans of the hybridized microarrays. Following quantile normalization of the data using a log_2_ ratio (R software package, version 3.1.2), we performed a low-intensity filtering. Only the circRNAs displaying ‘P’ or ‘M’ flags in the ‘All Targets Value’ field in at least 6/18 (33%) samples were kept for further analyses. Hierarchical clustering based on the ‘All Targets Value’ was conducted to display the circRNA expression patterns among the samples (Cluster and TreeView software by Eisen et al.; http://genome-www4.stanford.edu/MicroArray/SMD/restech.html).

Then, fold-change filtering and Student's *t*-testing were applied to identify differential circRNA between PTC tumors versus matching normal thyroid tissue as well as PTC tumors versus benign thyroid lesions. Specifically, only the circRNAs that exhibited fold-changes of greater than 2.0 and Student's *t*-test *p*-values of less than 0.05 were identified as differential circRNAs. Scatter plots were used to assess the variation in circRNA expression between each pairwise comparison, and volcano plots were used to visualize the significantly differential circRNAs between each pairwise comparison.

### Quantitative Real-Time Reverse-Transcription Polymerase Chain Reaction (qRT-PCR)

Following RNA extraction from PTC tumors and matching contralateral normal thyroid tissue (n = 6 pairs), cDNA was synthesized with a reverse transcriptase according to the kit’s instructions (SuperScript First-Strand Synthesis System for RT-PCR, Invitrogen, Carlsbad, CA, USA). circRNA expression was measured through qPCR (SYBR Green PCR Master Mix, Applied Biosystems, Foster City, CA, USA). PCR was conducted in a 20-μl reaction volume consisting of the following: 6.8 μl cDNA, 10 μl 2 x SYBR Green mix, 0.8 μl primer forward (5 μM), 0.8 μl primer reverse (5 μM), and 1.6 μl H_2_O. The qPCR reaction was performed on an ABI 7300 Real-Time PCR System (Applied Biosystems) as follows: one 2-min cycle at 50°C, one 10-min cycle at 95°C, forty cycles of 15 s at 95°C and 1 min at 60°C. β-actin served as a control. PCR reactions were conducted in triplicate. Relative circRNA expression was calculated with the 2^−ΔΔCt^ method.

### Prediction of circRNA-miRNA interactions

Circular RNAs have been shown to affect miRNA-mediated regulation of gene expression through miRNA sequestration [10]. Therefore, Arraystar miRNA target prediction software (Arraystar), which is based on TargetScan and miRanda algorithms [10], was applied to predict putative circRNA/miRNA interactions for the 12 differential circRNAs identified from the microarray and qRT-PCR validation experiments. In order to construct the circRNA-miRNA network, the Arraystar software was used to search for MREs on the 12 differential circRNAs, which were then used to identify putative miRNAs based on their seed-sequence complementarity [10]. The following parameters were used for this Arraystar MRE analysis as previously described [10]: seed type (seed-sequence complementarity) between nucleotides 2–7, proximity of AU-richness to seed sequence, and proximity to nucleotides pairing with the miRNA’s 3’ pairing sequence (nucleotides 13–16). Cytoscape 3.01 was then used to diagram the map of the circRNA/miRNA interaction network.

### Prediction of cancer-related circRNA-miRNA-target gene associations

Several online bioinformatics platforms were applied in conjunction in order to predict circRNA-miRNA-target gene associations. First, using the DIANA-miRPath v.3 platform [11], miRNA candidates were identified in KEGG cancer-related pathways within the circRNA-miRNA network. The following settings were applied on the DIANA-miRPath v.3 platform: KEGG analysis, TarBase 7.0, merge results by pathways union, FDR correction, a *p*-value threshold of 0.05, enrichment analysis method of Fisher’s exact test, and targeted pathways clusters/heatmap. Second, using our results from the Arraystar analysis and seed sequences derived from the miRBase database [12], we then matched the candidate circRNA(s) against the seed sequences of predicted target miRNA candidate(s) using the IntaRNA platform, which predicts interactions between RNA molecules [13]. The following settings were applied on the DIANA-miRPath v.3 platform: 0 suboptimal interactions, 5 minimum number of basepairs in seed, 0 maximum number of mismatches in seed, 37.0 temperature for energy computation, 22 folding window size target, and 22 max. basepair distance (target). After validating circRNA-target miRNA interaction, the DIANA-TarBase 7.0 database was then used to identify the top ten-ranking cancer-related target genes for each of the target miRNA candidates [14].

## Results

### Characterization of human thyroid samples

**Fig 1:**
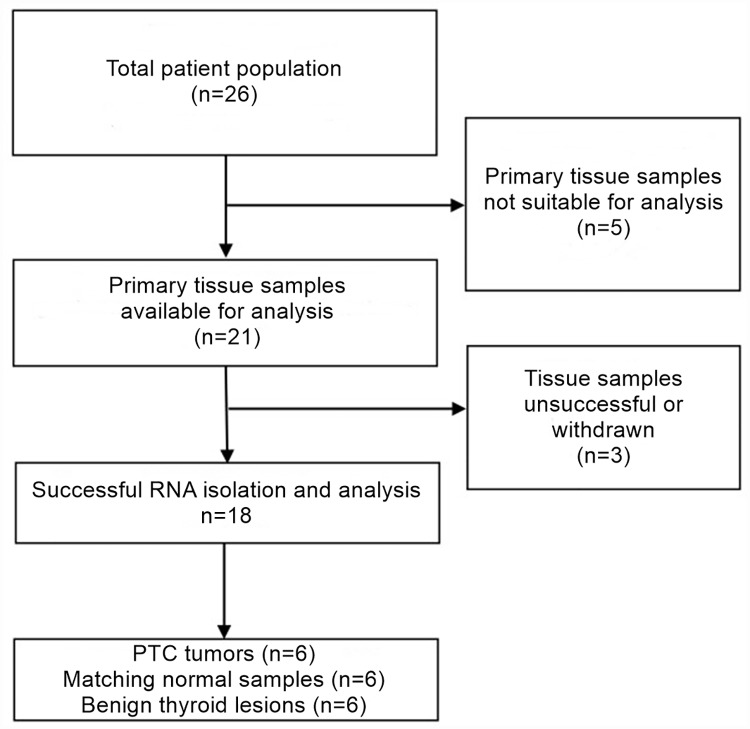
CONSORT flow diagram. CONSORT flow diagram detailing the selection of thyroid tissue samples. Tissue samples were not suitable for one of the following reasons: no tumor cells present in tumor samples (n = 3), only needle biopsy available (n = 2). Tissue samples were withdrawn for one of the following reasons: patients with bilateral cancer (n = 2), analysis unsuccessful (n = 1).

**Fig 2:**
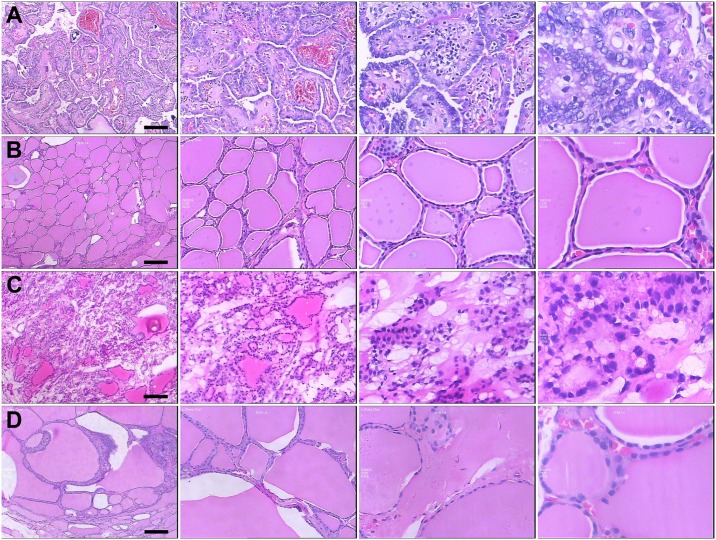
Immunohistochemical staining of thyroid specimens. Representative H&E-stained images of (A) PTC tumor samples, (B) contralateral normal thyroid tissue samples, (C) follicular adenoma samples, and (D) multinodular goiter samples. From left to right, image magnifications of 40×, 100×, 200×, and 400× are displayed. Scale bar = 50 μm.

On the basis of histopathological vetting, a total of 18 thyroid samples–consisting of six PTC tumors, six matching contralateral normal samples, and six benign thyroid lesions (i.e., three follicular adenoma samples and three multinodular goiter samples)–were finally included in the study. The CONSORT flow diagram detailing the selection of thyroid tissue samples is provided in Fig 1. The demographic and clinical characteristics of the included patients are provided in Table 1. Representative images of these H&E-stained thyroid specimens are provided in Fig 2.

**Table 1:** Demographic and clinical characteristics of included patients.

Group	Sample ID	Age (yrs)	Sex	Previous personal history of thyroid cancer or goiter?	Family history of thyroid cancer or goiter?
PTC	BG111B	43.0	F	None	None
Normal thyroid tissue	BG111N				
PTC	BG131B	40.5	F	None	None
Normal thyroid tissue	BG131N				
PTC	BG145B	42.2	M	None	Thyroid cancer (sister), goiter (mother)
Normal thyroid tissue	BG145N				
PTC	BG127B	41.1	M	None	None
Normal thyroid tissue	BG127N				
PTC	BG146B	38.1	F	Goiter	None
Normal thyroid tissue	BG146N				
PTC	BZ088DL1	45.4	F	None	None
Normal thyroid tissue	BZ088NL1				
Follicular adenoma	BG009N	42.5	M	None	None
Follicular adenoma	BG122B	38.1	F	None	None
Follicular adenoma	BZ064DR1	44.9	F	None	None
Multinodular goiter	BG068B	38.9	F	None	None
Multinodular goiter	BG100B	43.5	F	None	None
Multinodular goiter	BG115B	40.6	M	None	None

### Validation of thyroid samples’ RNA quality

Total RNA was extracted from all thyroid samples, and spectrophotometry was applied to assess RNA quality. All thyroid samples displayed OD260/OD280 ratios between 1.87 and 2.03 as well as OD260/OD230 ratios of greater than 2.02, indicating suitable RNA quality (S1 Fig). Moreover, electrophoresis revealed suitable RNA integrity and no observable evidence of DNA contamination (S2 Fig).

### Identification of differential circRNA profiles

**Fig 3:**
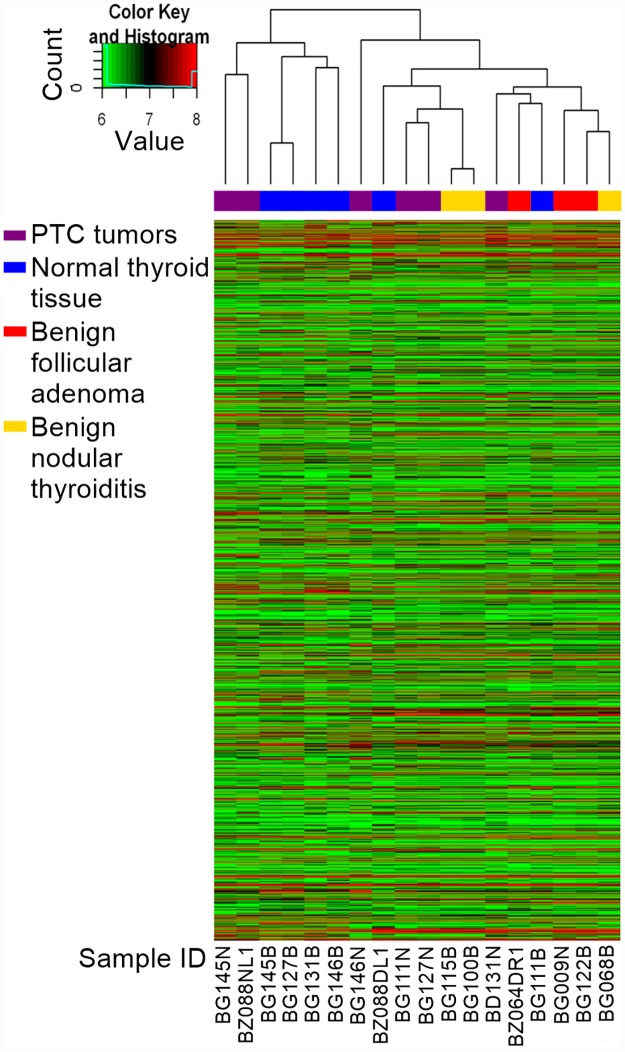
Hierarchical clustering of circRNA expression data. Hierarchical clustering of the circRNA expression data based on ‘All Targets Value’–which arranges the samples into groups based on their expression levels–revealed a distinguishable circRNA expression profiling among samples.

In order to profile circRNA expression in the thyroid samples, the Arraystar Human circRNA Microarray was used to perform sample labeling and microarray hybridization. Following low-intensity filtering, hierarchical clustering was conducted to display the circRNA expression patterns among the samples (Fig 3, S1 File).

**Fig 4:**
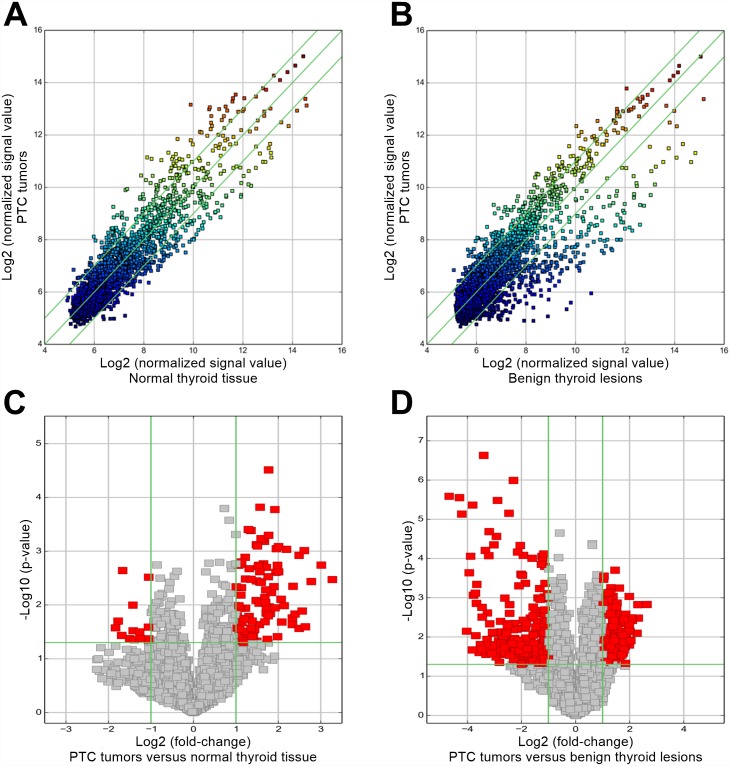
Scatter plots and volcano plots to identify differentially-expressed circRNAs. Scatter plots used to identify differentially-expressed circRNAs in (A) PTC tumors versus normal thyroid tissue and (B) PTC tumors versus benign thyroid lesions. The axis represent the mean normalized circRNA signal values for each comparator group (log_2_ scaled). The green fold-change lines represent 2.0× fold-changes, so the circRNAs lying above and below these green lines displayed greater than a 2.0-fold upregulation or downregulation. Volcano plots used to identify differentially-expressed circRNAs in (C) PTC tumors versus normal thyroid tissue and (D) PTC tumors versus benign thyroid lesions. The *x*-axis represents fold-change values (log_2_ scaled), while the *y*-axis represents *p*-values (-log_10_ scaled). The green vertical lines correspond to 2.0× upregulation and downregulation, respectively, while the green horizontal line corresponds to a *p*-value of 0.05. On this basis, the red rectangles represent the differentially-expressed circRNAs of statistical significance.

**Fig 5:**
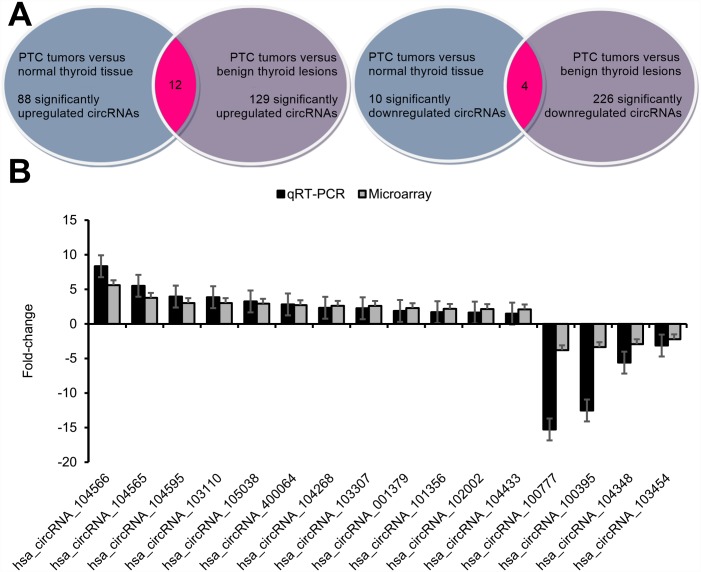
Identification and qRT-PCR validation of differential circRNAs. (A) There were 88 significantly upregulated circRNAs in PTC tumors versus normal thyroid tissue. There were 129 significantly upregulated circRNAs in PTC tumors versus benign thyroid lesions. Integrating these two comparisons, we found 12 overlapping significantly upregulated circRNAs in PTC tumors versus benign thyroid tissue. These 12 significantly upregulated circRNAs are detailed in Table 2. (B) There were 10 significantly downregulated circRNAs in PTC tumors versus normal thyroid tissue. There were 226 significantly downregulated circRNAs in PTC tumors versus benign thyroid lesions. Integrating these two comparisons, we found four overlapping significantly downregulated circRNAs in PTC tumors versus benign thyroid tissue. These four significantly downregulated circRNAs are detailed in Table 3. (C) qRT-PCR was used to validate the dysregulated expression of the 12 significantly upregulated circRNAs and the four significantly downregulated circRNAs. Data are displayed as means ± SEMs.

After applying fold-change filtering and Student's *t*-testing to identify differentially-expressed circRNAs (Fig 4), we identified 88 circRNAs that were significantly upregulated in PTC tumors as compared to normal thyroid tissue as well as 129 circRNAs that were significantly upregulated in PTC tumors as compared to benign thyroid lesions according to our pre-defined fold-change>2.0 and *p*-value<0.05 thresholds (Fig 5A, S2 File). A total of 12 significantly upregulated circRNAs were overlapping between the foregoing comparisons with hsa_circRNA_104566 (hsa_circ_0004458), hsa_circRNA_104565 (hsa_circ_0002111), hsa_circRNA_104595 (hsa_circ_0008016), and hsa_circRNA_103110 (hsa_circ_0004771) showing the highest fold-changes (in descending order; Fig 5A, Table 2). We also identified 10 circRNAs that were significantly downregulated in PTC tumors as compared to normal thyroid tissue as well as 226 circRNAs that were significantly downregulated in PTC tumors as compared to benign thyroid lesions according to our pre-defined fold-change<2.0 and *p*-value<0.05 thresholds (Fig 5A, S2 File). Four significantly downregulated circRNAs were overlapping between the foregoing comparisons (in descending order): hsa_circRNA_100777 (hsa_circ_0021553), hsa_circRNA_100395 (hsa_circ_0015278), hsa_circRNA_104348 (hsa_circ_0079891), and hsa_circRNA_103454 (hsa_circ_0067103) (Fig 5A, Table 3).

**Table 2:** Significantly upregulated circRNAs in PTC tumors versus benign thyroid tissue.

CircRNA	Alias	Fold-change	*P*-value	FDR	Predicted miRNA response elements (MREs)				
					MRE1	MRE2	MRE3	MRE4	MRE5
hsa_circRNA_104566	hsa_circ_0004458	↑5.600	0.0012	0.1653	hsa-miR-450b-5p	hsa-miR-205-3p	hsa-miR-95-5p	hsa-miR-329-5p	hsa-miR-19b-2-5p
hsa_circRNA_104565	hsa_circ_0002111	↑3.771	0.0002	0.1214	hsa-miR-450b-5p	hsa-miR-205-3p	hsa-miR-103a-2-5p	hsa-miR-95-5p	hsa-miR-329-5p
hsa_circRNA_104595	hsa_circ_0008016	↑3.016	0.0006	0.1559	hsa-miR-412-3p	hsa-miR-363-5p	hsa-miR-608	hsa-miR-564	hsa-miR-196b-3p
hsa_circRNA_103110	hsa_circ_0004771	↑3.015	0.0038	0.1997	hsa-miR-653-5p	hsa-miR-339-5p	hsa-miR-330-5p	hsa-miR-595	hsa-miR-629-3p
hsa_circRNA_105038	hsa_circ_0091894	↑2.925	0.0093	0.3269	hsa-miR-134-3p	hsa-miR-149-5p	hsa-miR-504-5p	hsa-miR-339-5p	hsa-miR-329-5p
hsa_circRNA_400064	hsa_circ_0092315	↑2.710	0.0285	0.4416	hsa-miR-658	hsa-miR-494-3p	hsa-miR-675-5p	hsa-miR-604	hsa-miR-361-3p
hsa_circRNA_104268	hsa_circ_0078738	↑2.616	0.0385	0.4893	hsa-miR-324-3p	hsa-miR-660-3p	hsa-miR-646	hsa-miR-214-3p	hsa-miR-628-5p
hsa_circRNA_103307	hsa_circ_0064557	↑2.603	0.0199	0.3949	hsa-miR-1264	hsa-miR-490-3p	hsa-miR-619-3p	hsa-miR-23a-3p	hsa-miR-23b-3p
hsa_circRNA_001379	hsa_circ_0000516	↑2.287	0.0439	0.5015	hsa-miR-18b-3p	hsa-miR-670-5p	hsa-miR-23a-5p	hsa-miR-125a-3p	hsa-miR-125b-5p
hsa_circRNA_101356	hsa_circ_0004846	↑2.178	0.0316	0.4489	hsa-miR-148b-5p	hsa-miR-330-3p	hsa-miR-29b-1-5p	hsa-miR-646	hsa-miR-660-5p
hsa_circRNA_102002	hsa_circ_0003505	↑2.147	0.0116	0.3308	hsa-miR-141-5p	hsa-miR-625-5p	hsa-miR-143-3p	hsa-miR-124-5p	hsa-miR-191-3p
hsa_circRNA_104433	hsa_circ_0081342	↑2.099	0.0103	0.3308	hsa-miR-497-5p	hsa-miR-545-3p	hsa-miR-636	hsa-miR-15a-5p	hsa-miR-335-3p

**Table 3:** Significantly downregulated circRNAs in PTC tumors versus benign thyroid tissue.

CircRNA	Alias	Fold-change	*P*-value	FDR	Predicted miRNA response elements (MREs)				
					MRE1	MRE2	MRE3	MRE4	MRE5
hsa_circRNA_100777	hsa_circ_0021553	↓3.807	8.33E-05	0.0116	hsa-miR-122-5p	hsa-miR-574-5p	hsa-miR-493-5p	hsa-miR-380-5p	hsa-miR-92a-2-5p
hsa_circRNA_100395	hsa_circ_0015278	↓3.358	0.0131	0.0913	hsa-miR-141-3p	hsa-miR-588	hsa-miR-660-3p	hsa-miR-136-5p	hsa-miR-200a-3p
hsa_circRNA_104348	hsa_circ_0079891	↓2.930	0.0200	0.1048	hsa-miR-519d-5p	hsa-miR-301a-3p	hsa-miR-611	hsa-miR-100-3p	hsa-miR-758-5p
hsa_circRNA_103454	hsa_circ_0067103	↓2.219	0.0230	0.1059	hsa-miR-591	hsa-miR-7-5p	hsa-miR-660-5p	hsa-miR-144-5p	hsa-miR-452-3p

### qRT-PCR validation of differential circRNAs

The 12 significantly upregulated circRNAs (Fig 5A, Table 2) as well as the four significantly downregulated circRNAs (Fig 5A, Table 3) were selected for qRT-PCR validation. The qRT-PCR results revealed that all 12 upregulated circRNAs and all four downregulated circRNAs were significantly differentiated between PTC tumors versus matching normal thyroid tissue (Fig 5B). The qRT-PCR-derived fold-changes mirrored those derived from the microarray data (Fig 5B).

### Construction of circRNA-miRNA network

**Fig 6:**
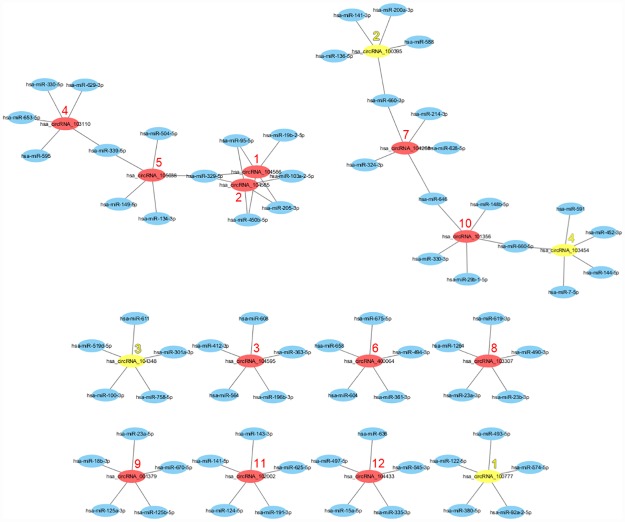
Network map of circRNA-miRNA interactions in PTC tumors. The network map consists of the previously identified 12 significantly upregulated circRNAs (represented by red nodes) and four significantly downregulated circRNAs (represented by yellow nodes) along with their 56 target miRNAs (represented by blue nodes). The numerical rank of each circRNA fold-change has been annotated next to each circRNA node.

As circRNAs interact with miRNAs via miRNA response elements (MREs), we searched for putative MREs through Arraystar's circRNA target prediction software. The predicted MREs for the significantly-upregulated circRNAs are listed in Table 2, with hsa-miR-329-5p being the most frequently targeted miRNA by three circRNAs (i.e., hsa_circRNA_104566, hsa_circRNA_104565, and hsa_circRNA_105038) followed by hsa-miR-450b-5p, hsa-miR-205-3p, hsa-miR-95-5p, hsa-miR-339-5p, and hsa-miR-646. The predicted MREs for the significantly-downregulated circRNAs are listed in Table 3, with no miRNA displaying a higher frequency of targeting. Based on the 16 dysregulated circRNAs and their predicted MREs, we constructed a network map of circRNA-miRNA interactions with Cytoscape (Fig 6).

### Prediction of cancer-related circRNA-miRNA-target gene associations

**Fig 7:**
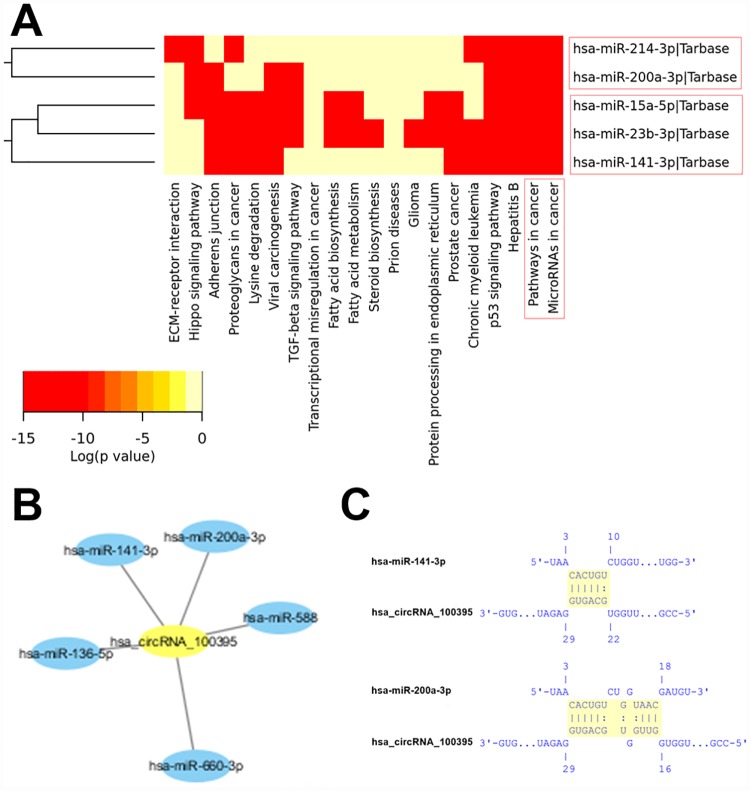
Prediction for circRNA-miRNA associations. (A) The targeted pathway heatmap analysis revealed significant correlations (depicted in red) between cancer-related pathways and two miRNA clusters: the miR-141-3p/miR-15a-5p/miR-23b-3p cluster and the miR-200a-3p/miR-214-3p cluster. The color-coded legend depicts the associated log-scaled *p*-values. (B) Based on the Arraystar-derived network map of circRNA-miRNA interactions, the candidate circRNA hsa_circRNA_100395 was predicted to interact with the two cancer-related miRNAs: hsa-miR-141-3p and hsa-miR-200a-3p. (C) Seed sequence matching predicts the direct interaction of hsa_circRNA_100395 with miR-141-3p and miR-200a-3p.

To further explore the underlying molecular mechanism(s) of the differential circRNAs, we first used the bioinformatics platform DIANA-miRPath v.3 to predict cancer-related circRNA-miRNA associations. From the initial list of 71 potential miRNAs identified from the aforementioned Arraystar analysis, the DIANA-miRPath platform was able to identify two cancer-related miRNA clusters within our pre-defined *p*-value<0.05 threshold: the miR-141-3p/miR-15a-5p/miR-23b-3p cluster and the miR-200a-3p/miR-214-3p cluster (Fig 7A, S3, S4 and S5 Figs). Comparing these two miRNA clusters to the Arraystar network map (Fig 6), we were able to identify that hsa_circRNA_100395 interacts with two miRNAs (miR-141-3p and miR-200a-3p) from the identified cancer-related miRNA clusters (Fig 7B). We then applied seed sequence matching on the IntaRNA platform to predict that hsa_circRNA_100395 has the potential to bind to both miR-141-3p and miR-200a-3p (Fig 7C). Finally, we searched the DIANA-TarBase database for the top-ranking cancer-related target genes for these two miRNAs (Tables 4 and 5). By comparing the lists of top-ranking cancer-related target genes, we were able to identify seven promising cancer-related genes that may be targets of the hsa_circRNA_100395/miR-141-3p/ miR-200a-3p axis in PTC (in order of descending rank): E2F3, GLS, CCND2, PTEN, CCNG1, CDC25B, and ZFPM2 (Tables 4 and 5).

**Table 4:** Top-ten ranking cancer-related target genes for hsa-miR-141-3p.

Gene Name	Gene Ensembl ID	Experimentally-Supported TarBase Methods	TarBase Predicted Score
**E2F3**	ENSG00000112242	Multiple	1.000
**TGFB2**	ENSG00000092969	Other	1.000
**GLS**	ENSG00000115419	HITS-CLIP	0.987
**CCND2**	ENSG00000118971	HITS-CLIP	0.984
**PRKCE**	ENSG00000171132	HITS-CLIP	0.982
**PTEN**	ENSG00000171862	Multiple	0.928
**CCNG1**	ENSG00000113328	Multiple	0.890
**CDC25B**	ENSG00000101224	Multiple	0.860
**GRB2**	ENSG00000177885	Multiple	0.755
**ZFPM2**	ENSG00000169946	Other	0.687

**Table 5:** Top-ten ranking cancer-related target genes for hsa-miR-200a-3p.

Target Gene	Gene Ensembl ID	Experimentally-Supported TarBase Methods	TarBase Predicted Score
**E2F3**	ENSG00000112242	Multiple	1.000
**GLS**	ENSG00000115419	HITS-CLIP	0.989
**CCND2**	ENSG00000118971	HITS-CLIP	0.985
**PTEN**	ENSG00000171862	Multiple	0.917
**CCNG1**	ENSG00000113328	Multiple	0.909
**CDC25B**	ENSG00000101224	HITS-CLIP	0.840
**ZFPM2**	ENSG00000169946	Other	0.714
**MCL1**	ENSG00000143384	HITS-CLIP	0.678
**MET**	ENSG00000105976	HITS-CLIP	0.647
**CCNE1**	ENSG00000105173	Multiple	0.491

## Discussion

In this study, we comprehensively profiled the circRNA expression of PTC tumors in order to improve our understanding of the pathogenesis of PTC as well as identify potential circRNA biomarkers for PTC. From microarray analysis followed by qRT-PCR validation, we found sixteen significantly differentiated circRNAs–twelve upregulated and four downregulated–in PTC tumors as compared to benign thyroid tissue. Applying a set of bioinformatics tools, we also constructed a network map of circRNA-miRNA interactions for these sixteen significantly differentiated circRNAs. Therefrom, we identified one promising downregulated circRNA in PTC tumors (hsa_circRNA_100395) that shows interactive potential with two cancer-related miRNAs (miR-141-3p and miR-200a-3p). From this analysis, we identified several promising cancer-related genes that may be targets of the dysregulated hsa_circRNA_100395/ miR-141-3p/miR-200a-3p axis in PTC tumors. These findings suggest that circRNA dysregulation may play a role in the pathogenesis of PTC and that several key circRNAs may show promise as candidate biomarkers for PTC. Moreover, our bioinformatics analysis suggests that the hsa_circRNA_100395/miR-141-3p/ miR-200a-3p axis may be involved in the pathogenesis of PTC.

PTC tumors–which account for approximately 80% of thyroid malignancies—are derived from the follicular cells residing in the thyroid gland’s epithelial layer [15]. Previous molecular research seeking to characterize PTC tumors has primarily revolved around genomic, epigenomic, and proteomic analyses [15–20]. More recently, researchers have investigated changes in the expression of mRNA transcripts as well as non-coding RNAs in PTC tumors, including miRNA and long non-coding RNA (lncRNA) transcriptomic analyses [21–26].

However, as circRNA-based research is an emerging area of investigation [9], no study to date has yet investigated circRNA expression in PTC tumors. So, here we comprehensively profiled the circRNA expression of PTC tumors by microarray analysis and found 12 significantly upregulated circRNAs as well as four significantly downregulated circRNAs in PTC tumors relative to benign thyroid tissue (Fig 5A). These 16 dysregulated circRNAs in PTC tumors were further validated by qRT-PCR testing (Fig 5B). Based on a comprehensive search of other circRNA studies in human malignancies, we found one significantly upregulated circRNA from this study—hsa_circRNA_104595 –that has also been shown to be significantly upregulated in prostate cancer cells [27]; however, all the other significantly dysregulated circRNAs did not cross-reference with any previously published literature on human cancers.

CircRNAs have been shown to negatively regulate the inhibitory effects of miRNAs upon their target mRNAs by directly binding to miRNAs via MREs, thereby essentially functioning as ‘miRNA sponges’ within the cellular RNA network [9]. Based on our Arraystar's circRNA target prediction analysis, we constructed a network map of circRNA-miRNA interactions for the sixteen significantly differentiated circRNAs in PTC tumors. In essence, this network map diagrams a cellular RNA network consisting of the 12 dysregulated circRNA nodes interacting with 71 miRNA nodes (Fig 6). In order to further investigate which miRNAs may be cancer-related, we applied several bioinformatics tools to identify one promising downregulated circRNA in PTC tumors (hsa_circRNA_100395) that shows interactive potential with two cancer-related miRNAs (miR-141-3p and miR-200a-3p) (Fig 7, S3, S4 and S5 Figs). Although we found no previous literature concerning hsa_circRNA_100395, there is substantial previous literature concerning the two miRNAs. Interestingly, both miR-141-3p and miR-200a-3p are members of the miR-200 family, which contains two miRNA clusters located on human chromosomes 1 and 12 (miRs-200a/b/429 and miRs-141/200c) [28]. With respect to the current study, both miR-141-3p and miR-200a-3p have been shown to regulate tumor growth and metastasis across several malignancies; however, the direction of miR-141-3p’s dysregulation is dependent upon the specific tissue type. For example, miR-141-3p and miR-200a-3p have been shown to be downregulated in breast cancer cells [29, 30], gastric cancer cells [31], and renal cell carcinoma cells [32, 33]. However, miR-141-3p has been shown to be upregulated in nasopharyngeal carcinoma cells [34], lung carcinoma cells [35], ovarian tumor cells [36], prostate tumor cells [37], and bladder cancer cells [38]. As we found that hsa_circRNA_100395 was significantly downregulated in PTC tumors here, our analysis suggests that miR-141-3p and/or miR-200a-3p should be consequently upregulated in PTC tumors as a result of regulatory disinhibition. This upregulation of miR-141-3p and/or miR-200a-3p may have effects on several cancer-related target genes that have been previously associated with these two miRNAs, including E2F3, GLS, CCND2, PTEN, CCNG1, CDC25B, and ZFPM2 (Tables 4 and 5). Further studies should focus on the dysregulation of the hsa_circRNA_100395/miR-141-3p/miR-200a-3p axis in PTC tumor cells in order to conclusively determine which cancer-related target genes are affected.

There are several limitations to this study. First, the sample size of this study was relatively small and limited to ethnic Han Chinese patients recruited from a single study site. Future studies should endeavor to recruit larger, more heterogeneous cohorts of patients in order to improve powering and reduce the influence of ethnogenetic biases. Second, our analysis was limited to thyroid tissue. Thus, other biosamples of interest–such as thyroid cyst fluid, plasma/serum, or urine–should be investigated in future studies. Third, due to the limited number of PTC samples, this study could not stratify the analysis on the basis of PTC grade or stage. As circRNA expression may vary with PTC grade or stage, future studies should collect larger numbers of PTC samples of varying grades and stages in order to analyze circRNA expression on these basis.

In conclusion, we found sixteen significantly differentiated circRNAs–twelve upregulated and four downregulated–in PTC tumors as compared to benign thyroid tissue. These findings suggest that circRNA dysregulation may play a role in the pathogenesis of PTC and that several key circRNAs may show promise as candidate biomarkers for PTC. Moreover, our bioinformatics analysis suggests that the hsa_circRNA_100395/miR-141-3p/miR-200a-3p axis may be involved in the pathogenesis of PTC.

## Supporting information

S1 Fig Spectrophotometric validation of RNA quality. Total RNA concentrations and quality were assessed by spectrophotometry. OD260/OD280 ratios between 1.8 and 2.1 and OD260/OD230 ratios of greater than 1.8 were deemed acceptable.
(TIF)

S2 Fig Electrophoretic validation of RNA integrity. RNA integrity and DNA contamination were assessed through electrophoresis on a denaturing agarose gel. The 28S and 18S ribosomal RNA bands were sharp, intense bands with the upper band approximately twice as intense as the lower band. The smaller, less intense bands represent low molecular weight RNAs (tRNA and 5S ribosomal RNA). DNA contamination—evidenced by high-molecular weight smearing above the 28S ribosomal RNA band–was not apparent. RNA degradation–evidenced by smearing of the ribosomal RNA bands–was not observed.
(TIF)

S3 Fig Complete targeted pathway heatmap. (TIF)

S4 Fig Complete pathway cluster dendogram. (TIF)

S5 Fig Complete miRNA cluster dendogram. (TIF)

S1 File CircRNA expression profiling data. (XLS)

S2 File Differentially-expressed circRNAs. (XLS)
