# Supplementary figures and images for: Microarray profiling of circular RNAs in human papillary thyroid carcinoma
Source: PLoS One. 2017 Mar 13;12(3):e0170287. doi: 10.1371/journal.pone.0170287 (PMC5347999; doi:10.1371/journal.pone.0170287)

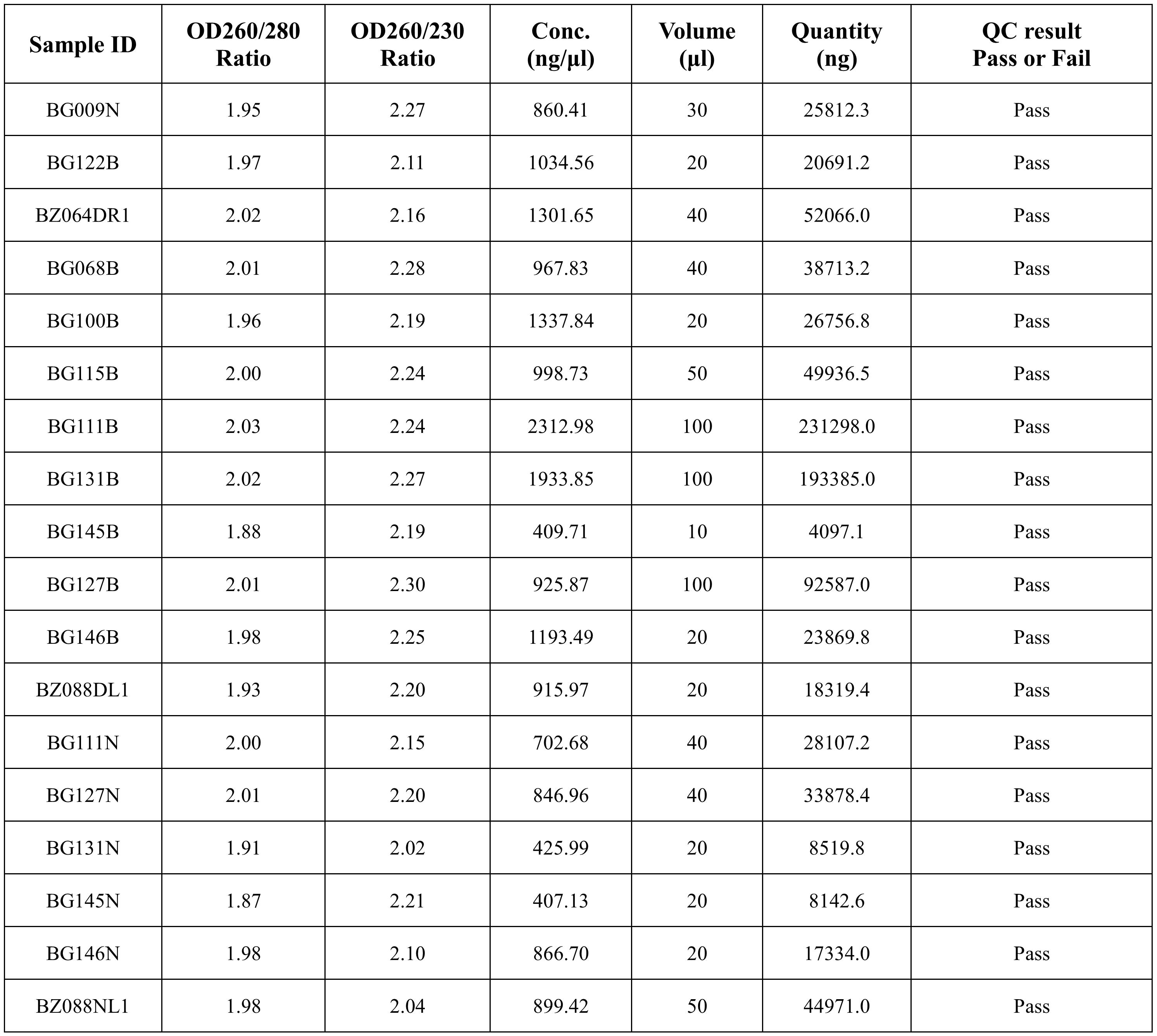

Supplement: S1 Fig — Total RNA concentrations and quality were assessed by spectrophotometry. OD260/OD280 ratios between 1.8 and 2.1 and OD260/OD230 ratios of greater than 1.8 were deemed acceptable. (TIF) [file pone.0170287.s001.tif]

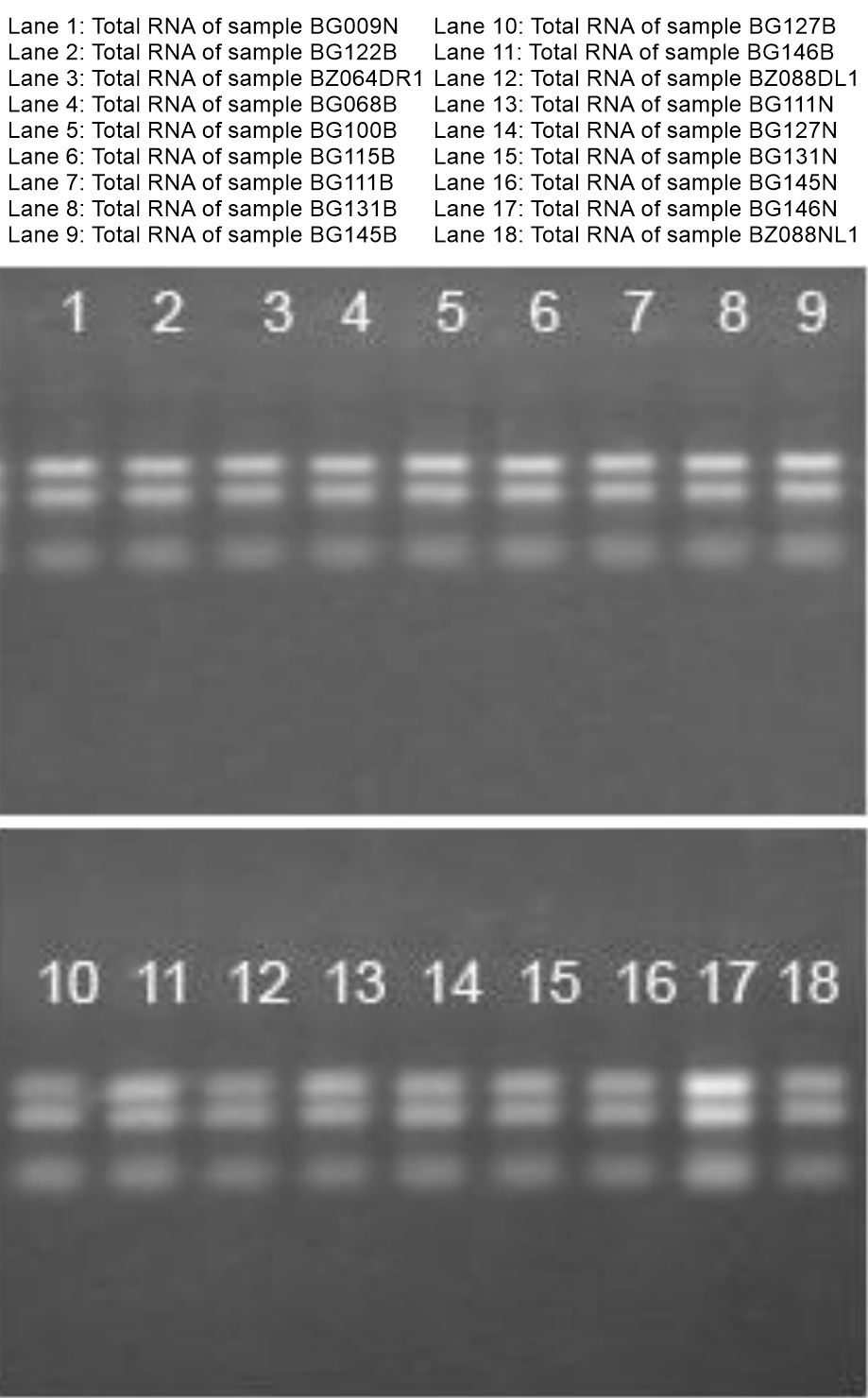

Supplement: S2 Fig — RNA integrity and DNA contamination were assessed through electrophoresis on a denaturing agarose gel. The 28S and 18S ribosomal RNA bands were sharp, intense bands with the upper band approximately twice as intense as the lower band. The smaller, less intense bands represent low molecular weight RNAs (tRNA and 5S ribosomal RNA). DNA contamination—evidenced by high-molecular weight smearing above the 28S ribosomal RNA band–was not apparent. RNA degradation–evidenced by smearing of the ribosomal RNA bands–was not observed. (TIF) [file pone.0170287.s002.tif]

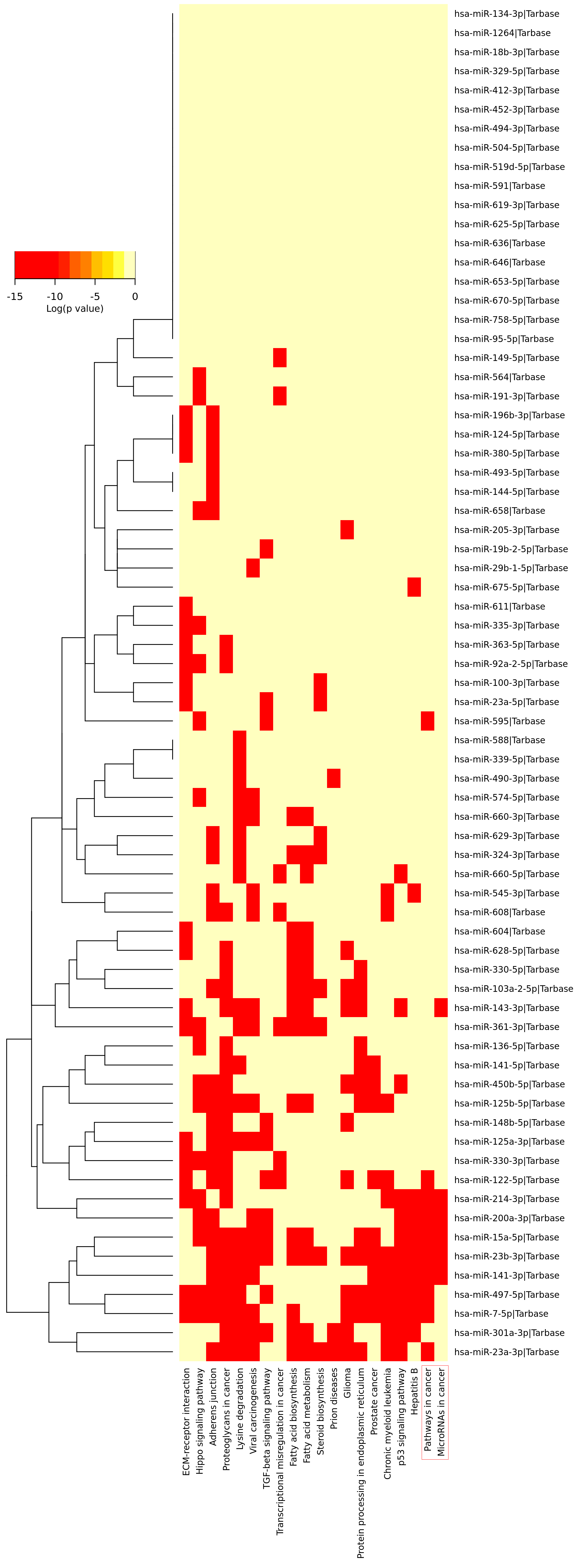

Supplement: S3 Fig — (TIF) [file pone.0170287.s003.tif]

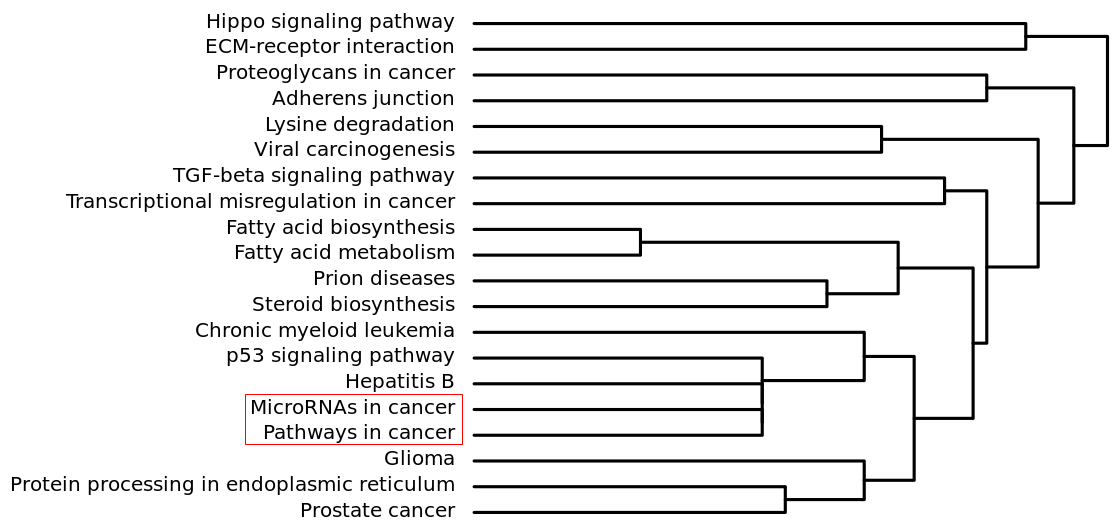

Supplement: S4 Fig — (TIF) [file pone.0170287.s004.tif]

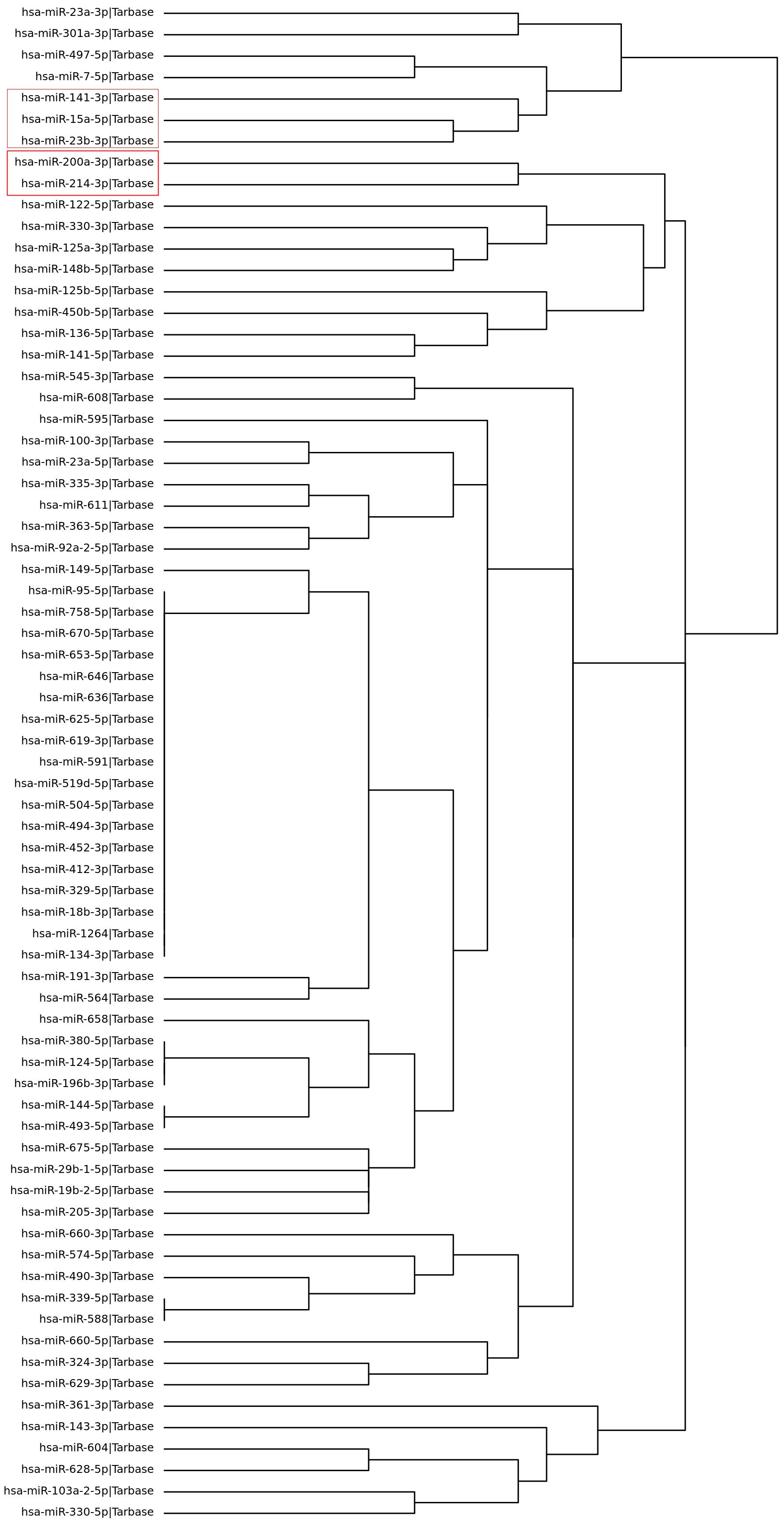

Supplement: S5 Fig — (TIF) [file pone.0170287.s005.tif]
